# Effects of boundary conditions on foot behaviour in the standing position in 3D finite element foot model

**DOI:** 10.1186/1757-1146-7-S1-A38

**Published:** 2014-04-08

**Authors:** Shane Johnson, Haihua Ou

**Affiliations:** 1 University of Michigan and Shanghai Jiao Tong University Joint Institute, Shanghai, 200240, China

## Introduction

The most common physical injuries are injuries of the lower extremity. In fact, controlled studies on highly physically active groups such as athletes and military personnel show that five injury types are repeatedly cited as accounting for over 50 percent of all training injuries: stress fractures, overuse injuries of the knee, Plantar Fasciitis, Achilles Tendonitis, and ankle sprains [1-5].

Three-dimensional finite element analysis (3D FEA) of the foot in the standing position allows researchers to analyze the relationship between foot behavior and orthotic designs, which may help to relieve or prevent such injuries. Various 3D FEA models of the foot in the standing position show very different boundary conditions, including: fixing the fibula and tibia at different points between the ankle and knee, fixing the talus, and applying slip/no-slip conditions in the articular surfaces [6-12]. This may have a large effect on overall foot stiffness and the strain of the Plantar Aponeurosis.

This study is developed to investigate the influence of these boundary conditions on the overall foot stiffness and strain in the Plantar Aponeurosis in the standing position.

## Method

A parametric study was conducted by varying the boundary conditions on a 3D FEA foot model by:

1. Varying slip/no slip conditions at the articular surfaces (Tibia/Talus, Fibula/Talus, Talus/Calcaneous, Talus/Navicular, Calcaneous/Cuboid);

2. Fixing/Pinning different points between the proximal and distal ends of the tibia and fibula;

3. Applying Achilles tendon forces of different magnitudes;

The strain of the Plantar Aponeurosis and plantar pressures under different boundary conditions are compared with experimental results found in the literature [13,14].

## Results

The result shows that changing boundary conditions has a large effect on the overall foot stiffness and strain in the Plantar Aponeurosis.

This analysis provides researchers conducting 3D finite element analysis on the foot with a guide on which parameters, especially the force-displacement boundary conditions, have the largest effect on particular foot behaviors. This is critical in later analyzing the interaction between the foot and new orthotic designs.
